# Hysterectomy and Bilateral Salpingoovariectomy in a Transsexual Subject without Visible Scaring

**DOI:** 10.1155/2010/845029

**Published:** 2010-08-01

**Authors:** Anna Myriam Perrone, Maria Cristina Scifo, Valentina Martelli, Paolo Casadio, Paolo Giovanni Morselli, Giuseppe Pelusi, Maria Cristina Meriggiola

**Affiliations:** ^1^Centre for Sexual Health, Gynecology and Obstretrics Unit, S. Orsola Hospital, University of Bologna, Via Massarenti 190, 40138 Bologna, Italy; ^2^Centre for Sexual Health, Plastic Surgical Unit, S. Orsola Hospital, University of Bologna, Via Massarenti 190, 40138 Bologna, Italy

## Abstract

*Objective*. To report on the use of laparoendoscopic single-site surgery (LESS) for the management of total hysterectomy (TH) with bilateral salpingoovariectomy (BSO) in a subject affected by gender identity disorder. *Design*. Case report. *Setting*. University Hospital. *Patient(s)*. A 27-year-old affected by Gender Identity Disorder underwent a hysterectomy and BSO as part of surgical sex reassignment. *Intervention(s)*. Laparoendoscopic single-site surgery access for TH and BSO. *Main Outcome Measure(s)*. The procedure was performed without incident. The trocar placement was easy and safe, without inadvertent port removal. No vascular or visceral injuries, loss of pneumoperitoneum, or intraoperative port site bleeding occurred. *Result(s)*. A detailed description of the technique of a single-site surgery for management of hysterectomy and BSO. *Conclusion*. Our case presents the first report of single-site surgery for surgical treatment of subjects affected by GID.

## 1. Introduction

Since 1989 when Reich performed the first laparoscopic Hysterectomy, laparoscopic surgery has become the standard treatment for gynecologic diseases. Major advantages of the laparoscopic approach are less blood loss and a shorter hospital stay compared to hysterectomy via abdominal access [1, 2].

Recently, an even less invasive alternative to conventional laparoscopy or robotic surgery has been developed: laparoendoscopic single-site surgery (LESS), also known as single-port surgery. With the LESS approach the entire operative intervention is performed through one single small incision, and the scar can be hidden in the umbilicus [3–5].

Single port laparoscopy is an attempt to further enhance the cosmetic benefits of minimally invasive surgery while minimizing the potential morbidity associated with multiple incisions [6].

Preliminary advances in LESS as applied to urologic [7] and gastrointestinal surgery [8] demonstrate that the technique is feasible provided that optimal surgical technical expertise, advanced skills, and optimal instrumentation are available.

In the field of gynaecology, the literature presents a case of ectopic pregnancy [9], a report on three ovarian cysts [10], and a preliminary study on laparoscopic-assisted vaginal hysterectomy (LAVH) [11]. The authors conclude that LESS offers cosmetic advantages, shorter recovery, and no short-term or long-term complications compared with the standard laparoscopic approach [9–11].

To our knowledge, there are no published reports of total hysterectomy and bilateral salpingoovariectmy (BSO) perfomed by using the LESS approach in subjects affected by Gender Identity Disorder (GID). We chose a women affected by GID for our first approach at LESS: these subjects are biological females that view themselves as males (commonly called Female to Male, FtM) and undergo hormonal treatment and surgery in order to adjust their bodies to the gender to which they feel they belong. These subjects are routinely submitted to hysterectomy and bilateral salpingoovariectomy (BSO) as part of sex reassignment.

The purpose of this paper is to describe the technique that we used, to assess the feasibility of hysterectomy and BSO by using a laparoendoscopic single-site trocar through transumbilical access.

## 2. Case

A 27-year-old affected by GID was submitted to hysterectomy and BSO as part of the surgical sex reassignment. The subject was nullipara and virgo. Her BMI was 25.7 (kg/m^2^). No relevant comorbidities or history of previous abdominal surgeries was recorded. She had received hormonal therapy with testosterone gel (50 mg die) for two years, until the day before surgery. Transabdominal ultrasonography showed normal uterus diameters and structures and normal ovarian volumes.

The surgery was performed by an experienced surgeon in laparoendoscopic hysterectomy (about 50 procedures) but who had performed only one previous LESS procedure; the first was an ovarian endometriotic cyst.

The patient was under general endotracheal anesthesia, in lithotomic position. A uterine manipulator to move the uterus was placed (Hohl manipulator, Storz).

**Figure 1:**
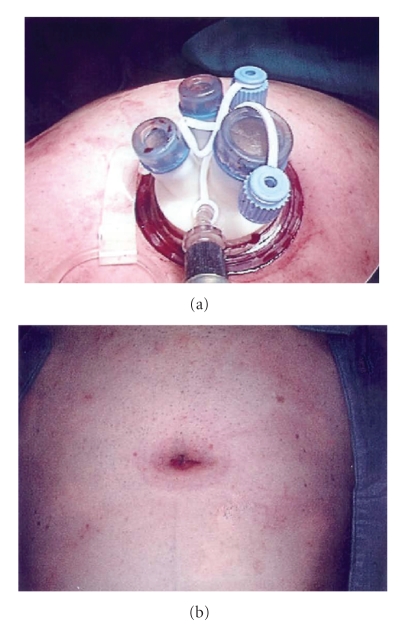
(a) The trocar (tri-port) composed of two ports for CO2 Insufflations/desufflations and three operative accesses (2 of 5 mm for the instrumentation and 1 of 12 mm generally for the optic) placed into the umbilical site. (b) Invisible scar in the umbilicus at the end of the surgical procedure.

The surgical procedure was performed with a single incision, and a single trocar (Laparo Endoscopic Single-Site Surgery; Olympus Winter & IBE GMBH, Hamburg, Germany) was placed into the umbilical site (Figure 1(a)). This new device consisted of three components: the introducer, the fixing valve, and the trocar itself. A removal ribbon is provided to remove the device from the incision at the end of the procedure. The trocar (called tri-port) is composed of two ports for CO2 insufflations/desufflations and three operative accesses (two of 5 mm for the instrumentation and one of 12 mm generally for the optic). Each port is equipped with a special gel valve so that the surgeon may safely change the instruments without interrupting the surgery. Before the surgical procedure the device was prepared, and the fixing valve was inserted into the end of the introducer.

We performed pneumoperitoneum with a Verres needle until 14 mmHg. A routine incision of 1 cm with a scalpel was performed inside the umbilicus before inserting the 10 mm multiuse trocar. This procedure allows quick access to the abdomen and reduces the length of the incision. The trocar was removed, and we inserted the new device passing the introducer through the incision while pushing gently. When the distal end of the introducer reached the abdominal cavity we pressed the thumb switch to eject the distal ring and to remove the introducer from the incision ensuring that the distal ring was inside the abdomen. Pulling the sleeve up, the distal and the proximal rings pair off. The sleeve was cut to expose the valves and was connected to the insufflations with one of the venting ports. A small puncture of the valves was performed with a scalpel to give the instruments access. Finally we introduced a videolaparoscope 0 degree 5 mm (Olympus) and two instruments that had been previously lubricated. For the hysterectomy and BSO we used an ultracision (Ethicon Ultracision G110 Harmonic Scalpel ESU Generator), graspers, scissors, bipolar coagulator, and suction/irrigation. The operative surgeon and the camera assistant stood on the patient's right and left side, respectively. Changes in the position of the instruments and optics were carried out according to the needs of the surgeon.

We started the procedure with the division of left and right round ligament. The window was opened in the large ligament; then vesicouterine space was opened, and a bladder dissection was performed. For these surgical steps scissors, a bipolar coagulator, and graspers were used according to the needs of the surgeon.

The uterine pedicle was prepared, cauterized, and dissected by ultracision. Then, the suspensor ligament of the ovary was grasped and cauterized with ultracision. The valve of the manipulator was inserted into the vagina, and it was easily opened with the ultracision. Once the uterus had been freed its extraction was easy through colpotomy. Closure of the vagina was performed using the vaginal route. Finally, we pulled out the distal ring through the incision to remove the device. The trocar placement was easy and safe, without inadvertent port removal. Operation time defined from the first incision to the final vaginal suture was 90 minutes. Estimated blood loss defined by usage of suction was 20 mL. No vascular or visceral injuries, loss of pneumoperitoneum, or intraoperative port site bleeding occurred. After surgery, there were no wound hematomas, wound infections, or delayed bleeding. The patient conveyed complete satisfaction regarding the cosmetic result (Figure 1(b)) and postoperative pain control. She was discharged on day 2 with only optional analgesic therapy.

## 3. Discussion

This is the first paper in literature of total laparoscopic hysterectomy performed in a GID patient with one port laparoscopic access. Our experience shows that hysterectomy with LESS is possible, feasible, and safe.

The scar hidden deep in the umbilical fold may represent a great advancement in the sex reassignment surgery, leading to practical and psychological advantages for the GID patient.

Although in the literature, an open access has been described [9–11], in our experience this was not necessary, and the standard laparoscopic technique that uses the Verres needle to perform the pneumoperitoneum and then the introduction of the LESS trocar was used. The access is easy, fast and does not require a learning curve if the surgeon is experienced in performing laparoscopy. As previously described, major limits of this technique are represented by the small space between the instruments both outside and inside the abdominal cavity [9, 10]. The surgeon is limited in his movements and the camera assistant quite easily crosses the surgeon's instruments. Due to these problems, the use of a 5-mm 30° telescope, the combination of long and short instruments, curved handgrip instruments that increase the space outside the abdomen, and the use of a flexible camera on the tip have been advised to facilitate the surgery and to reduce the operation time [10]. In this operation we did not use the 30° telescope because in previous attempt we found that it does not facilitate the procedure. Most likely this is because we are trained with a 0° telescope, and the shift to the 30° telescope requires more training. We completed the entire procedure using standard laparoscopic instruments. Therefore, although the development of such instruments specific to this technique is advisable, in our experience this was not absolutely necessary. It has also been hypothesized that a four-port trocar for the use of another instrument could be developed. Although in some steps of hysterectomy such as the dissection of the vesicouterine space a fourth instrument could be useful, this port would certainly complicate the procedure by increasing the crossing of the instruments. In our experience the use of laparoscopic instruments such as the ultracision, which allows for tissue fusion/vessel sealing, spot coagulation, and endoscissor functions by the same instrument, is more important than the fourth laparoscopic access.

New endoscopic access without visible scarring represents an important advancement in particular for the sex reassignment surgery of FtM both for practical and psychological aspects. From a practical point of view, the preservation of the possibility to use the abdominal flap for a future phalloplasty may represent an important advantage for those GID patients who wish to complete their transition. Some authors have reported more complications due to scaring such as necrotic areas on the neopenis as results of skin scars from previous incisions. Moreover, scarring can compromise the sensitivity of the skin due to the sectioning of some coetaneous nerves. If the area used from abdominal flap does not include any scars, the skin sensation and erogenous sensation in the glands can be well preserved [12]. From a psychological point of view, scars are important for social interactions, including the responses of strangers and others who can see the scars. Some researchers have confirmed the importance of social interactions on scar-related behaviours.

Wolszon argued that physical appearance influences social interactions because it is a readily available source of information about a person [13]. The absence of pelvic scars, which would identify to the person and to others the type of surgery they have undergone, may help complete the full integration of these female subjects into the new male role. For these subjects, the presence of the scars in particular sites of the body represents the stigmata of pelvic surgery, particularly hysterectomy. This is not only a cosmetic problem but the sign of the correlation between the subjective experience (to combat the female image and female stigmata) and the objective reality of the scar (their hysterectomy).

In conclusion we think this is an interesting and promising technique that certainly needs further evaluation in clinical trials, preferably compared with standard laparoscopy, so that efficacy, safety, and potential benefits can be evaluated. The cost of this device is about 500 Euros, that is equal to the cost of the three monouse trocars in the traditional laparoscopy.

We believe that it has the greatest potential application in particular operations such as the sex reassignment surgery that we described here.
